# Aligning Sequences by Minimum Description Length

**DOI:** 10.1155/2007/72936

**Published:** 2008-01-02

**Authors:** JohnS Conery

**Affiliations:** 1 Department of Computer and Information Science, University of Oregon, Eugene, OR 97403, USA

## Abstract

This paper presents a new information theoretic framework for aligning sequences in bioinformatics. A transmitter compresses a set of sequences by constructing a regular expression that describes the regions of similarity in the sequences. To retrieve the original set of sequences, a receiver generates all strings that match the expression. An alignment algorithm uses minimum description length to encode and explore alternative expressions; the expression with the shortest encoding provides the best overall alignment. When two substrings contain letters that are similar according to a substitution matrix, a code length function based on conditional probabilities defined by the matrix will encode the substrings with fewer bits. In one experiment, alignments produced with this new method were found to be comparable to alignments from . A second experiment measured the accuracy of the new method on pairwise alignments of sequences from the BAliBASE alignment benchmark.

## 

[1,2,3,4,5,6,7,8,9,10,11,12,13,14,15,16,17,18,19,20,21,22,23,24,25,26,27,28,29,30,31,32,33,34,35,36,37,38,39,40]

## References

[B1] Myers EW The fragment assembly string graph Bioinformatics 2005 21 suppl. 2 ii79 ii85 16204131 10.1093/bioinformatics/bti1114

[B2] Altschul SF Madden TL Schaffer AA Gapped BLAST and PSI-BLAST: a new generation of protein database search programs Nucleic Acids Research 1997 25 17 3389 3402 10.1093/nar/25.17.3389 9254694 PMC146917

[B3] Phillips AJ Homology assessment and molecular sequence alignment Journal of Biomedical Informatics 2006 39 1 18 33 10.1016/j.jbi.2005.11.005 16380300

[B4] Wrabl JO Grishin NV Gaps in structurally similar proteins: towards improvement of multiple sequence alignment Proteins 2004 54 1 71 87 14705025 10.1002/prot.10508

[B5] Sjölander K Phylogenomic inference of protein molecular function: advances and challenges Bioinformatics 2004 20 2 170 179 10.1093/bioinformatics/bth021 14734307

[B6] Webb B-JM Liu JS Lawrence CE BALSA: Bayesian algorithm for local sequence alignment Nucleic Acids Research 2002 30 5 1268 1277 10.1093/nar/30.5.1268 11861921 PMC101229

[B7] Rissanen J Modelling by the shortest data description Automatica 1978 14 5 465 471 10.1016/0005-1098(78)90005-5

[B8] Grünwald P A minimum description length approach to grammar inference Connectionist, Statistical, and Symbolic Approaches to Learning for Natural Language Processing, Lecture Notes in Computer Science 1996 1040 Springer, Berlin, Germany 203 216

[B9] Brazma A Jonassen I Vilo J Ukkonen E Honavar V, Slutski G Pattern discovery in biosequences International Conference on Grammar Inference (ICGI '98), Lecture Notes in Artificial Intelligence 1998 1433 Springer, Ames, Iowa, USA 257 270

[B10] Cai L Malmberg RL Wu Y Stochastic modeling of RNA pseudoknotted structures: a grammatical approach Bioinformatics 2003 19 suppl. 1 i66 i73 12855439 10.1093/bioinformatics/btg1007

[B11] Searls DB The computational linguistics of biological sequences Artificial Intelligence and Molecular Biology, Menlo Park, Calif, USA 1993 American Association for Artificial Intelligence 47 120

[B12] Bsearls D Linguistic approaches to biological sequences Computer Applications in the Biosciences 1997 13 4 333 344 9283748 10.1093/bioinformatics/13.4.333

[B13] Bairoch A PROSITE: a dictionary of sites and patterns in proteins Nucleic Acids Research 1992 20 2013 2018 1598232 10.1093/nar/20.suppl.2013 PMC333978

[B14] Vingron M Waterman MS Sequence alignment and penalty choice. Review of concepts, case studies and implications Journal of Molecular Biology 1994 235 1 1 12 10.1016/S0022-2836(05)80006-3 8289235

[B15] Henikoff S Scores for sequence searches and alignments Current Opinion in Structural Biology 1996 6 3 353 360 10.1016/S0959-440X(96)80055-8 8804821

[B16] Giribet G Wheeler WC On gaps Molecular Phylogenetics and Evolution 1999 13 1 132 143 10.1006/mpev.1999.0643 10508546

[B17] Nozaki Y Bellgard M Statistical evaluation and comparison of a pairwise alignment algorithm that a priori assigns the number of gaps rather than employing gap penalties Bioinformatics 2005 21 8 1421 1428 10.1093/bioinformatics/bti198 15591359

[B18] Reese JT Pearson WR Empirical determination of effective gap penalties for sequence comparison Bioinformatics 2002 18 11 1500 1507 10.1093/bioinformatics/18.11.1500 12424122

[B19] Allison L Wallace CS Yee CN Finite-state models in the alignment of macromolecules Journal of Molecular Evolution 1992 35 1 77 89 10.1007/BF00160262 1518085

[B20] Schmidt JP An information theoretic view of gapped and other alignments Proceedings of the 3rd Pacific Symposium on Biocomputing (PSB '98), Maui, Hawaii, USA, January 1998 561 572 9697212

[B21] Aynechi T Kuntz ID An information theoretic approach to macromolecular modeling: I. Sequence alignments Biophysical Journal 2005 89 5 2998 3007 10.1529/biophysj.104.054072 16254389 PMC1366797

[B22] Morgenstern B DIALIGN 2: improvement of the segment-to-segment approach to multiple sequence alignment Bioinformatics 1999 15 3 211 218 10.1093/bioinformatics/15.3.211 10222408

[B23] Brudno M Chapman M Göttgens B Batzoglou S Morgenstern B Fast and sensitive multiple alignment of large genomic sequences BMC Bioinformatics 2003 4 66 10.1186/1471-2105-4-66 14693042 PMC521198

[B24] Schneider TD Information content of individual genetic sequences Journal of Theoretical Biology 1997 189 4 427 441 10.1006/jtbi.1997.0540 9446751

[B25] Krasnogor N Pelta DA Measuring the similarity of protein structures by means of the universal similarity metric Bioinformatics 2004 20 7 1015 1021 10.1093/bioinformatics/bth031 14751983

[B26] Conery JS Realign: grammar-based sequence alignment University of Oregon, http://teleost.cs.uoregon.edu/realign

[B27] Dayhoff MO Schwartz RM Orcutt BC A model of evolutionary change in proteins Atlas of Protein Sequence and Structure, Washington, DC, USA, 1978 5 suppl. 3 345 352

[B28] Mount DW Bioinformatics: Sequence and Genome Analysis 2004 2 Cold Spring Harbor Laboratory Press, New York, NY, USA

[B29] Henikoff S Henikoff JG Amino acid substitution matrices from protein blocks Proceedings of the National Academy of Sciences of the United States of America 1992 89 22 10915 10919 10.1073/pnas.89.22.10915 1438297 PMC50453

[B30] Gonnet GH Cohen MA Benner SA Exhaustive matching of the entire protein sequence database Science 1992 256 5062 1443 1445 10.1126/science.1604319 1604319

[B31] Karlin S Altschul SF Methods for assessing the statistical significance of molecular sequence features by using general scoring schemes Proceedings of the National Academy of Sciences of the United States of America 1990 87 6 2264 2268 10.1073/pnas.87.6.2264 2315319 PMC53667

[B32] Eddy SR Where did the BLOSUM62 alignment score matrix come from? Nature Biotechnology 2004 22 8 1035 1036 10.1038/nbt0804-1035 15286655

[B33] Aurrecoechea C Heiges M Wang H ApiDB: integrated resources for the apicomplexan bioinformatics resource center Nucleic Acids Research 2007 35 D427 D430 10.1093/nar/gkl880 17098930 PMC1669770

[B34] Thompson JD Higgins DG Gibson TJ CLUSTAL W: improving the sensitivity of progressive multiple sequence alignment through sequence weighting, position-specific gap penalties and weight matrix choice Nucleic Acids Research 1994 22 22 4673 4680 10.1093/nar/22.22.4673 7984417 PMC308517

[B35] Thompson JD Plewniak F Poch O A comprehensive comparison of multiple sequence alignment programs Nucleic Acids Research 1999 27 13 2682 2690 10.1093/nar/27.13.2682 10373585 PMC148477

[B36] Carter R Speculations on the origins of Plasmodium vivax malaria Trends in Parasitology 2003 19 5 214 219 10.1016/S1471-4922(03)00070-9 12763427

[B37] Cline M Hughey R Karplus K Predicting reliable regions in protein sequence alignments Bioinformatics 2002 18 2 306 314 10.1093/bioinformatics/18.2.306 11847078

[B38] Conery JS Lynch M Nucleotide substitutions and the evolution of duplicate genes Proceedings of the 6th Pacific Symposium on Biocomputing (PSB '01), Big Island of Hawaii, Hawaii, USA, January 2001 167 178 10.1142/9789814447362_0018 11262937

[B39] Pearson WR Comparison of methods for searching protein sequence databases Protein Science 1995 4 6 1145 1160 7549879 10.1002/pro.5560040613 PMC2143149

[B40] Hulo N Bairoch A Bulliard V The PROSITE database Nucleic Acids Research 2006 34 D227 D230 10.1093/nar/gkj063 16381852 PMC1347426

